# Gorlin syndrome associated with small bowel carcinoma and mesenchymal proliferation of the gastrointestinal tract: case report and review of literature

**DOI:** 10.1186/1471-2407-10-360

**Published:** 2010-07-07

**Authors:** Peter M Prodinger, Mario Sarbia, Jörg Maßmann, Christian Straka, Günther Meyer, Ortrud K Steinlein

**Affiliations:** 1 Chirugische Klinik München-Bogenhausen, Denninger Str. 44, 81679 Munich, Germany; 2 Gemeinschaftspraxis Pathologie, Lachnerstr. 2, 80639 Munich, Germany; 3 Interne Klinik Dr. Argirov, Münchner Str. 23-29, 82335 Berg, Germany; 4 Institut für Humangenetik, University of Munich, University Hospital, Goethestr. 29, 80336 Munich, Germany

## Abstract

**Background and Case Presentation:**

A patient with nevoid basal cell carcinoma syndrome (Gorlin syndrome) presented with two unusual clinical features, i.e. adenocarcinoma of the small bowel and extensive mesenchymal proliferation of the lower gastrointestinal tract.

**Conclusions:**

We discuss the possibility that these two features are pathogenetically linked to the formerly undescribed patient's *PTCH *germ line mutation.

## Background

Nevoid basal cell carcinoma syndrome (NBCCS), also known as Gorlin syndrome, is a rare autosomal dominant disorder caused by mutations in the Patched (*PTCH*) gene on chromosome 9q22. NBCCS patients often have a coarse facial appearance with macrocephaly, frontal bossing and prognathism. Falx calcification is frequently found in affected individuals and skeletal anomalies such as bifid ribs, wedge-shaped or fused vertebra and thumb deformities are common. Multiple keratocysts of the jaw can develop between childhood and young adulthood, and most patients get their first basal cell carcinoma (BCC) in their early 20s [1]. Although some additional tumor types such as medulloblastoma, cardiac and ovarian fibromas, and lipomas are known to have an increased frequency in NBCCS, intestinal tumors are not known to be part of the clinical phenotype [2]. In the following we describe the clinical, histopathological and genetic findings in a patient showing a combination of an unusual phenotype of NBCCS, a rare adenocarcinoma of the ileum and mesenchymal proliferation of the small bowel and probably also the stomach.

### Case presentation

#### Medical history

**Figure 1:**
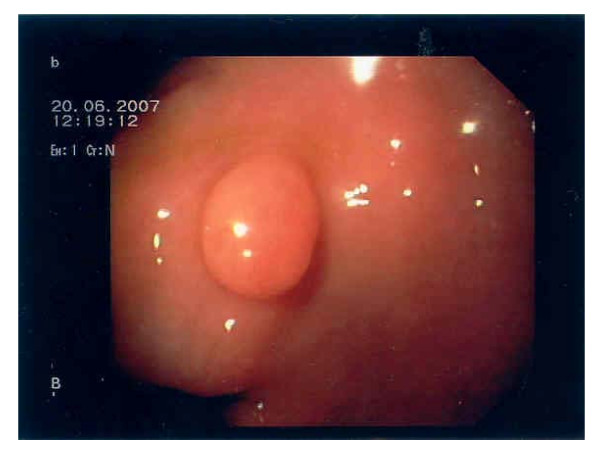
**Endoscopy of upper GI-tract**. Polyploid structure seen in the antrum.

The patient, a Caucasian male, had multiple BCCs surgically removed from the his face, requiring extensive facial skin graft repair, at the age of 49. At the age of 52 years he presented with weight loss and sub-ileus. A CT-scan indicated a stenosing process located proximal to the ileocolic valve. Consecutively explorative laparotomy was performed and showed an obstructing tumor mass at the terminal ileum that required ileocecal resection extended to the regional lymph nodes of the mesentery. Due to unusual histopathological findings esophagogastroduodenoscopy and colonoscopy were conducted after the operation and showed polyploid structures in the stomach (Figure 1) and the neoterminal ileum. Endoscopic mucosal biopsies were taken from the colon, ileum, duodenum and stomach.

**Figure 2:**
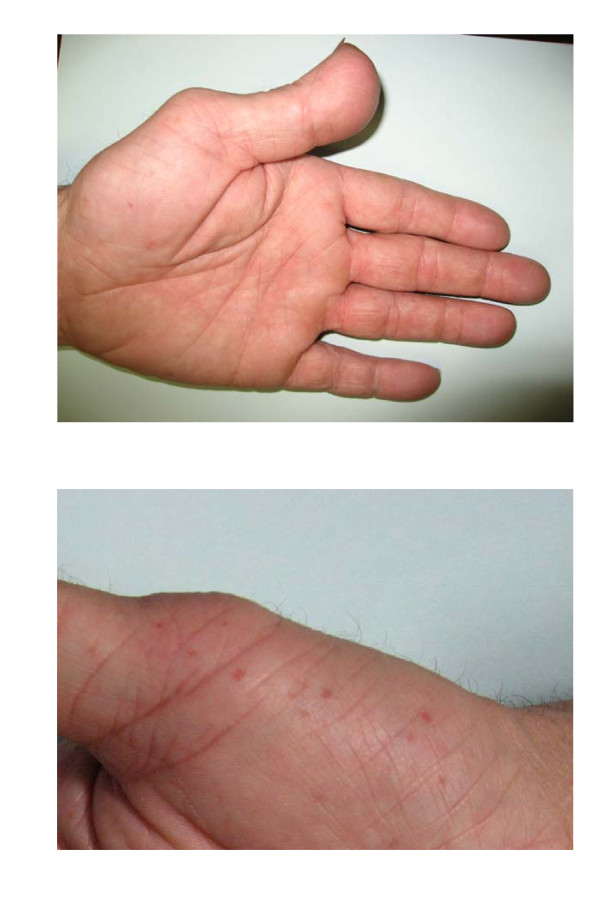
**Patient's hands**. **(a) **Left hand: broad thumb, mild brachydactyly, palmar pits. **(b) **Right hand: multiple palmar pits.

At the same time several skin tumors from the patients shoulder were diagnosed as BCCs, which finally led to the suspicion of NBCCS. Additionally both hands showed multiple palmar pits and mild brachydactyly, the left thumb was enlarged and deformed (Figure 2). Jaw cysts and intracerebral calcifications were excluded by MRI scan. Although family history was empty for BCC and other tumors, the combination of observed alterations of the hands and multiple BCCs was strongly suggestive of NBCCS and *PTCH *gene screening was initiated.

#### Pathological findings

**Figure 3:**
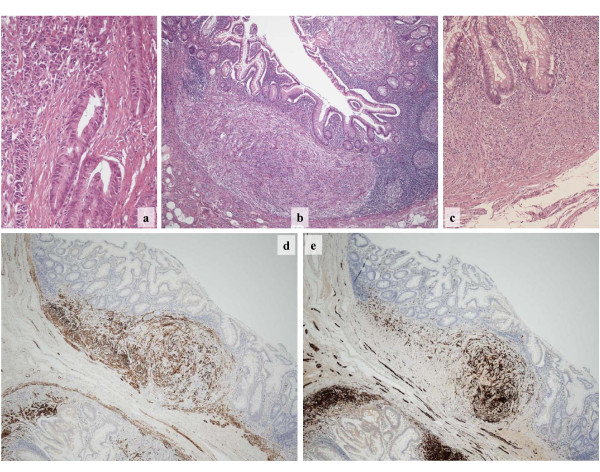
**Small intestine histopathology**. **(a)**: Poorly differentiated adenocarcinoma composed of malignant glands (bottom, right) and diffusely distributed cancer cells (top, left). **(b)**: Low power magnification of small intestine mucosa showing two separate spindle cell nodules in the deep mucosa. **(c)**: Higher magnification of a spindle cell nodule shows bland cytological features of the tumor cells. **(d)**: Immunostaining for desmin highlights the lamina muscularis mucosae as well as approximately half of the tumor cells. **(e)**: Immunostaining of the same area for s100 decorates nerve fibres in the submucosa as well as the rest of the tumor cells.

Macroscopic examination of the operation specimen from the terminal ileum showed an ulcerated tumor with a maximum diameter of 4.5 cm. Microscopic examination revealed a poorly differentiated adenocarcinoma composed of glandular and signet ring cell elements (Figure 3a). The carcinoma infiltrated into the mesenterial fatty tissue and showed lymphatic vessel invasion. Seven out of 33 regional lymph nodes presented metastatic involvement. The TNM classification was: pT3 pN2 (7/33) cM0 R0 L1 V0 G3 [3]. Immunohistochemical analysis revealed no evidence for neuroendocrine differentiation of the carcinoma, i.e. no expression of synaptophysin and chromogranin A. The signet ring component of the carcinoma showed an unusual high proliferative index, as determined by MIB-1 expression, of 90%. The proliferative index of the glandular component was 50%.

Additionally, the entire non-carcinoma bearing small bowel showed a coarse nodular appearance of the luminal surface with intact covering mucosa. After, the nodules were located in the mucosa and submucosa of the small intestine with a maximum nodule diameter of 3.5 cm. Macroscopic examination revealed numerous nodules (>100) that centred at the muscularis mucosa and extended into the submucosa and the lamina propria of the mucosa. The nodules were characterized by unsharp delineation without forming capsules (Figure 3b). They were composed of intermingling spindle cells with scant cytoplasm and bland nuclei (Figure 3c) that seemed to originate from the muscularis mucosae. The spindle cells appeared homogeneous and no mitotic activity was visible. The latter was confirmed by the nearly complete absence of MIB-1 staining. The proliferative index was below 1%. Immunohistochemical analysis revealed an intimate mixture of smooth muscle cells characterized by strong expression of desmin (Figure 3d) and smooth muscle actin and Schwann cells that expressed S100 (Figure 3e). Antibodies against neurofilament proved the presence of single neurons, indicating the proliferation of both, Schwann cells and neuronal cells within the nodules. No expression of CD117 or CD34 was detectable in the spindle cell tumors arguing against the presence of a gastrointestinal stromal tumor (GIST). Staining with Cathepsin D, Synaptophysin and Chromogranin A revealed only single positive cells, excluding ganglioneuromatosis.

The composition of smooth muscle cells, Schwann cells and neuronal cells and the multifocal occurrence within the small bowel did not fit into any known histopathological classification. These findings are best described as mesenchymal proliferation with neural and smooth muscle components.

These changes were also found in the endoscopic biopsies taken from the terminal ileum and duodenum but not in those from the colon. Microscopic examination of the stomach-biopsies showed chronic gastritis due to H. pylori infection. One biopsy taken in the antrum of the stomach also showed the unusual spindle cell accumulations described above, but only in small areas close to the section border and without confirmation of neuronal components. Thus the diagnosis of mesenchymal proliferation with neural and smooth muscle components was only tentative for the nodules present in the stomach. Consultation of the Department of Pathology of Basel/Switzerland (Dr. E. Bruder) confirmed our findings.

#### Mutation screening

**Figure 4:**
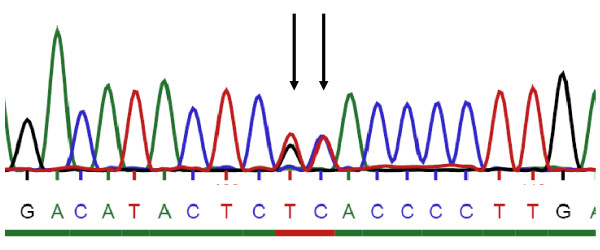
**Results of sequencing**. Heterozygous stop codon mutation (c.1136_1137AC>GA; p.Y379X) found in exon 8 is shown. Mutated nucleic acids are indicated by arrows.

Sequencing of all 23 coding exons and adjacent intronic sequences of the PTCH gene led to the detection of a heterozygous stop codon mutation (c.1136_1137AC>GA; p.Y379X) in exon 8. The adjacent nucleotides adenine and cytosine in position 1136 and 1137 of the *PTCH *gene were mutated into guanine and adenine, respectively, changing a codon for tyrosine into a stop codon. This mutation has not been described before. It can be expected to completely abolish PTCH function, with serious consequences for the sonic hedgehog signalling pathway. The mutation confirmed the clinical diagnosis of NBCCS in our patient (Figure 4).

## Discussion

The NBCCS patient described here displays two unusual clinical features, small bowel adenocarcinoma and extensive mesenchymal proliferation with neural and smooth muscle components of the small bowel. Endoscopic examination and histopathological studies of biopsies provided an educated guess that the stomach was involved too. Although the small bowel represents 75% of the length and 90% of the surface of the GI-tract, tumors of the small bowel account only for 2% of all GI-malignancies. The rarity of the respective conditions strongly argues against an independent occurrence of both, small bowel carcinoma and NBCCS in our patient. Given the odds it is much more likely that a causative relation between the carcinoma and the *PTCH *germ line mutation exists. The same argument holds true for the mesenchymal proliferation present in the patient's GI tract. It is more likely that this previously not described finding is causally linked to the mutation in the tumor suppressor gene *PTCH *than that it represents the outcome of an entirely independent pathogenetic chain of events.

The PTCH protein acts in a negative feedback pathway as a receptor for different hedgehog proteins [4]. As a key regulator of embryonic development hedgehog signalling is involved in cell growth and differentiation in a wide variety of tissues and organs. The *PTCH *germ line mutations in NBCCS patients are either inherited from an affected parent or due to *de novo *events in a parental germ cell. The truncating germ line mutation found in our patient is likely to abolish the function of one copy of PTCH. However, it can not be completely excluded that a severely truncated form of the protein is produced that might interfere with the normal PTCH gene product, causing some kind of clinical phenotype. Tumorigenesis starts if inactivation of the second, normal *PTCH *allele by either loss-of-heterozygosity or point mutation occurs by chance in a single cell (two-hit mutagenesis). The resulting reactivation of the hedgehog/PTCH pathway not only causes basal cell carcinoma, but also contributes to the formation of tumors such as medulloblastoma and rhabdomyosarcoma [5]. Furthermore, several recent studies found evidence for an involvement of hedgehog signalling and PTCH in tumorigenesis of the GI- tract. Animal studies have shown that hedgehog signalling is crucial for the normal development of the gut [6]. Reactivation of this pathway is therefore likely to start uncontrolled cell growth, and it has already been demonstrated that hedgehog signalling is widely active in sporadic gut-derived tumors [7]., Ligand-dependent activation of the hedgehog/PTCH pathway by various external stimuli such as injury of the GI epithelium by acid, alcohol or *Helicobacter pylori *infection has been identified as the main pathomechanism underlying this observation [8]. The coexistence of a PTCH germ line mutation and a rare small bowel adenocarcinoma in our patient might indicate that a two-hit mutational inactivation of *PTCH *is a second though probably rare pathomechanism in GI malignancies.

A highly unusual finding in our patient were the spindle-cell masses in the small bowel that displayed a high degree of cellular inhomogenity. Leiomyomatosis in the GI-tract has been reported occasionally in syndromes such as neurofibromatosis type 1 and tuberous sclerosis, but without the neural cell component found in our patient [9-11]. It is therefore tempting to speculate that reactivation of the PTCH/hedgehog pathway might have induced the growth of spindle cells in the GI-tract of our patient. The variability of cell types present can be explained by the important role of sonic hedgehog signalling in the activation and differentiation of progenitor cells. Hedgehog signalling has shown to promote the development of neural crest cells that are capable of multilineage differentiation. These cells give rise not only to neurons and glial cells but also to different types of mesenchymal cells including smooth muscle and connective tissue cells [11]. This role in the development of multipotent progenitor cells fits with the coexistence of neural and smooth muscle cells seen in the immunohistochemical analysis of the spindle-cell nodules.

## Conclusion

Several questions remain: does our patient just represent an exotic case or is mesenchymal proliferation with neural and smooth muscle components of the small bowel a so far unknown clinical feature of NBCCS? Are both the mesenchymal proliferation and the small bowel carcinoma pathogenetically linked to the *PTCH *stop codon mutation? The fact that mesenchymal proliferation of the small bowel has not been previously reported in NBCCS does not necessarily exclude it from the list of possible complications. As long as it remains asymptomatic such feature is likely to remain unrecognized because the small bowel is not included in routine diagnosis of the GI-tract. If the adenocarcinoma in our patient is indeed pathogenetically connected with the mesenchymal proliferation, the malignant potential of the later is probably too low to raise a red flag in a syndrome this rare. More patients with this rare familial tumor syndrome need to be clinically evaluated to see if the mesenchymal proliferation in the small bowel should be added to the phenotypic spectrum of NBCCS.

### Consent

Written informed consent was obtained from the patient for publication of this case report and any accompanying images. A copy of the written consent is available for review by the Editor-in-Chief of this journal.

## Competing interests

The authors declare that they have no competing interests.

## Authors' contributions

PP and OS drafted and wrote the paper. PP, OS and MS designed the figures. JM, CS and GM corrected the paper. PP, GM and MS provided surgical and pathological guidance. All authors were involved in treatment and diagnosis of the patient and finalized and approved the manuscript.

## Pre-publication history

The pre-publication history for this paper can be accessed here:

http://www.biomedcentral.com/1471-2407/10/360/prepub
